# Oscillations in Cerebral Haemodynamics in Patients with Falciparum Malaria

**DOI:** 10.1007/978-1-4614-4989-8_15

**Published:** 2012-07-21

**Authors:** Christina Kolyva, Hugh Kingston, Ilias Tachtsidis, Sanjib Mohanty, Saroj Mishra, Rajya Patnaik, Richard J. Maude, Arjen M. Dondorp, Clare E. Elwell

**Affiliations:** 1 Department of Medical Physics and Bioengineering, University College London, Malet Place Engineering Building, Gower Street, London, WC1E 6BT UK; 2 Mahidol-Oxford Tropical Medicine Research Unit, Mahidol University, Bangkok, Thailand; 3 Department of Internal Medicine, Ispat General Hospital, Rourkela, India; 4 Centre for Tropical Medicine, University of Oxford, Oxford, UK

**Keywords:** Cerebral hemodynamics, Falciparum malaria

## Abstract

Spontaneous oscillations in cerebral haemodynamics studied with near-infrared spectroscopy (NIRS), become impaired in several pathological conditions. We assessed the spectral characteristics of these oscillations in 20 patients with falciparum malaria admitted to Ispat General Hospital, Rourkela, India. Monitoring included continuous frontal lobe NIRS recordings within 24 h of admission (*Day 0*), together with single measurements of a number of clinical and chemical markers recorded on admission. Seven patients returned for follow-up measurements on recovery (*FU*). A 2,048 sampling-point segment of oxygenated haemoglobin concentration ([ΔHbO_2_]) data was subjected to Fourier analysis per patient, and power spectral density was derived over the very low frequency (VLF: 0.02–0.04 Hz), low frequency (LF: 0.04–0.15 Hz) and high frequency (HF: 0.15–0.4 Hz) bands. At *Day 0*, VLF spectral power was 21.1 ± 16.4, LF power 7.2 ± 4.6 and HF power 2.6 ± 5.0, with VLF power being statistically significantly higher than LF and HF (*P* < 0.005). VLF power tended to decrease in the severely ill patients and correlated negatively with heart rate (*r* = 0.57, *P* < 0.01), while LF power correlated positively with aural body temperature (*r* = 0.49, *P* < 0.05). In all but one of the patients who returned for *FU* measurements, VLF power increased after recovery. This may be related to autonomic dysfunction in severe malaria, a topic of little research to date. The present study demonstrated that application of NIRS in a resource-poor setting is feasible and has potential as a research tool.

## Introduction

Falciparum malaria is a major public health problem in the developing world. It is caused by the protozoan parasite *Plasmodium falciparum* and transmitted to humans when an infected female *Anopheles* mosquito takes a blood meal, injecting sporozoites into the bloodstream as a by-product. Sporozoites multiply inside hepatocytes for an average of 6 days before releasing around 10^5^ merozoites into the bloodstream. These mature and multiply inside red blood cells (RBCs) during a 48-h incubation period after which the infected RBCs burst, enabling the offspring from each burst cell to infect around 8–10 new RBCs. With a total parasite burden of 10^7^ to 10^8^ the patient becomes febrile with flulike symptoms, while severe disease with involvement of multiple organs can develop when the parasite number exceeds 10^11^ to 10^12^. At some point, often 1 week after presentation, some of the merozoites divide into male and female gametocytes that are taken up when mosquitoes aspirate blood from an infected subject, closing the life cycle of the parasite [1].

Patients infected by *P. falciparum* experience additional adverse symptoms, compared to other malaria species, due to the unique feature of this particular parasite to cause infected RBCs to adhere to the capillary and venular endothelium of various organs, especially the brain. This sequestration leads to reduced microvascular flow, which is further aggravated by the reduced deformability of an infected subject’s RBCs [2].

Near-infrared spectroscopy (NIRS) has been previously used to investigate tissue oxygenation and metabolism in the adult brain [3]. Due to its portable, low-cost and non-invasive nature, it lends itself to environments with limited resources in terms of clinical monitoring. NIRS provides valuable information not only through the magnitudes of the measured parameters but also as a trend measurement. Such measurements in healthy adults have revealed slow oscillations in cerebral NIRS recordings [4], which are known to be impaired by pathological conditions like Alzheimer’s disease [5], cerebral microangiopathy [6] and cerebral infarction [7]. Their frequencies are distinctly below the heart rate and respiration frequencies, they are spontaneous and their origin is controversial [4].

The aim of the present study was to assess the spectral characteristics of the spontaneous oscillations in transcranial NIRS recordings in patients with malaria and on recovery and to investigate the mechanism causing them.

## Methods

Twenty non-sedated patients (14 males; age range 19–70 years) with asexual *P. falciparum* parasitaemia were studied at the Ispat General Hospital, Rourkela, India. Severe malaria was defined according to the modified Hien criteria [8] and cerebral malaria was defined as a Glasgow coma score (GCS) of <11. The study was approved by the Ispat General Hospital Ethics Committee and written informed consent was obtained from all patients or their families. All patients were receiving standard antimalarial treatment at the time of study, according to current guidelines [17].

Physical examination of the patient was performed on admission (*Day 0*) and a venous blood sample was taken to confirm the diagnosis by detection of asexual stage parasites on a peripheral blood film. The term *Day 0* is used throughout this study to indicate the first 24 h of admission. With the patient lying in the supine position and resting, frontal lobe continuous wave NIRS recordings were obtained at 6 Hz during *Day 0* over the course of at least 30 min (NIRO 300, Hamamatsu Photonics). The source–detector separation was 4 cm. Pulse oximetry measurements were simultaneously obtained at 1 Hz (Radical-7, Masimo). Seven of the 20 patients who consented to provide data in *Day 0* returned for follow-up measurements on recovery (*FU*), at a median of 15 days after *Day 0*.

Initial patient evaluation upon admission included single records of clinical parameters such as aural body temperature, GCS, mean blood pressure, heart rate (HR) and arterial oxygen saturation, and chemical markers, such as alanine transaminase, bilirubin, creatine kinase, total haemoglobin, base excess and lactate. From the NIRS measurements and assuming a differential pathlength factor of 6.26 [9], concentration changes of oxygenated [ΔHbO_2_] and deoxygenated haemoglobin [ΔHHb] were derived according to the modified Beer–Lambert law, as well as tissue oxygenation index (TOI) by application of the spatially resolved spectroscopy technique. Pulse oximetry provided measures of oxygen saturation, HR, methaemoglobin, total haemoglobin and plethysmography variability index (an index of the variability of the plethysmographic waveform).

All NIRS-derived data were resampled every 0.16 s (6.25 Hz). For each patient, a 327.68 s (=2,048  ×  0.16 s) data segment was selected and only this selection was included in the analysis described from this point onwards. The selection was based first upon identifying 2,048-point segments during which the standard deviation of the corresponding TOI data was less than 10% of the mean value (as a way of selecting data with as little noise as possible). Linear regression analysis was then performed on these segments (TOI vs. time) and the segment with the slope closest to zero was selected for further analysis (as a way of selecting data with stable TOI). [ΔHbO_2_] and [ΔHHb] data were linearly detrended and averages for TOI and oximetry data were calculated. Because spontaneous oscillations are most prominent in the [ΔHbO_2_] signal [4], the [ΔHbO_2_] data were run through a fast Fourier transform algorithm, without prior filtering. From the derived power spectral density (PSD), the spectral power of three frequency bands was determined, in line with previous analysis [10]: 0.02–0.04 Hz (very low frequency; VLF), 0.04–0.15 Hz (low frequency; LF) and 0.15–0.4 Hz (high frequency; HF). Spectral power was defined as the area under the PSD curve over a given frequency band, divided by the corresponding frequency range (0.02, 0.11 and 0.25 Hz for the VLF, LF and HF spectral power, respectively), in order to render valid comparisons of spectral power between different frequency bands. For noise reduction purposes, the spectral power of each frequency band was then normalised with the spectral power of the whole PSD curve.

Statistical significance was set to *P*  < 0.05. Means were compared with Student’s  *t*-tests and multiple testing was accounted for via the Bonferroni correction. Linear regression analysis was used to investigate relations between different parameters.

## Results

Table 15.1 summarises the baseline characteristics of the patients. Five patients had cerebral, nine non-cerebral severe and six uncomplicated falciparum malaria.

**Table 15.1:** Patient demographics on admission

	Cerebral	Non-cerebral severe	Uncomplicated
*n*	5	9	6
Age	25 (24–25)	45 (22–70)	44 (19–65)
Body temperature (°C)	37.6 (35.5–39.4)	37.3 (35.9–39.4)	37.3 (36.5–38.6)
GCS	7 (4–9)	13 (11–15)	15 (15–15)
Systolic blood pressure (mmHg)	133 (125–145)	118 (102–147)	125 (105–139)
Diastolic blood pressure (mmHg)	80 (63–105)	76 (59–96)	74 (65–92)
Heart rate (bpm)	130 (98–149)	101 (75–135)	93 (74–101)
Haemoglobin (g/dl)	9.0 (4.8–13)	9.8 (5.8–13.5)	12.9 (9.3–15.5)
Creatinine (mg/dl)	2.32 (1.9–2.8)	2.09 (1–4.5)	1.07 (0.7–1.6)
Total bilirubin (mg/dl)	8.6 (0.7–27.2)	5.2 (0.4–24.5)	1.3 (0.4–2)

Table entries are mean (range)

**Fig. 15.1:**
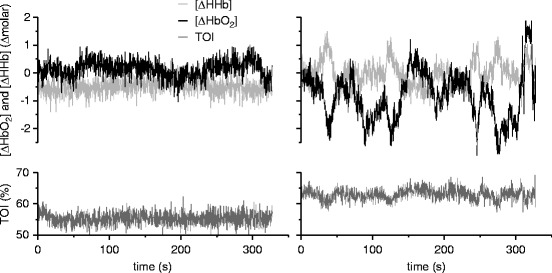
Concentration changes of oxygenated [ΔHbO_2_] and deoxygenated haemoglobin [ΔHHb] (*top*) and TOI (*bottom*) in patients with cerebral (*left*) and uncomplicated (*right*) malaria

*Day 0*: The spectral power of the VLF band was statistically significantly higher than that of the LF and HF bands (Table 15.2). Cases with cerebral malaria tended to have lower spectral power of the VLF band compared to non-cerebral severe cases and uncomplicated cases (Table 15.2). There was no difference in TOI between malaria groups and overall TOI was 58.1  ±  6.8% (mean  ± SD). Figure 15.1 shows examples of NIRS data from two age-matched patients, a case with cerebral malaria who did not survive and an uncomplicated case who made a full recovery, with striking differences in the oscillatory pattern of the signals.

**Table 15.2:** *Day 0* slow oscillation spectral characteristics

	All	Cerebral	Non-cerebral	Uncomplicated
Norm. VLF power	21.1 ± 16.4	8.8 ± 6.8	21.2 ± 13.9	31.3 ± 20.1
Norm. LF power	7.2 ± 4.6*	8.0 ± 6.1	6.8 ± 3.6	7.0 ± 5.5
Norm. HF power	2.6 ± 5.0*^,^**	2.4 ± 3.3	3.7 ± 7.0*	1.1 ± 0.7*
TOI (%)	58.1 ± 6.8	58.1 ± 5.4	59.5 ± 8.5	55.9 ± 5.4

*VLF* very low frequency, *LF* low frequency, *HF* high frequency, *TOI* tissue oxygenation index

*P  <  0.05 comparing VLF to LF or HF; **P  <  0.05 comparing LF to HF

Table entries are mean  ±  SD

Linear regression analysis was implemented in order to investigate possible relationships between VLF, LF and HF spectral power with all clinical parameters recorded upon admission and during *Day 0*. Oximetry data were available only in 13 of the 20 patients who participated in the study. The strongest correlation for VLF spectral power was with heart rate on admission (*Y*  =  −0.42*X* +  65.88, *r*  =  0.572, *P*  <  0.01, *n*  =  20) and the strongest correlation for LF power was with body temperature (*Y*  =  1.97*X*  −  66.32, *r*  =  0.488, *P*  < 0.05, *n*  =  20).

**Fig. 15.2:**
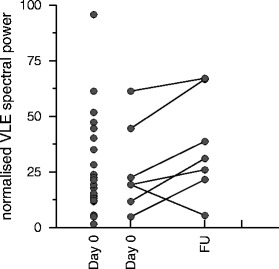
VLF spectral power in patients with falciparum malaria, on the day of admission (*Day 0*) and after recovery (*FU*). The spectral power of the VLF band increased upon recovery in all but one of the patients for whom follow-up measurements were available

*Follow-up*: The spectral power of the VLF band increased after recovery in six of the seven patients for whom follow-up measurements were available (Fig. 15.2), on average from 26.2  ±  19.8 to 36.7  ±  23.0 (*P*  =  NS). No obvious pattern was observed for the corresponding changes in LF and HF spectral power.

## Discussion and Conclusions

Our study on the spectral characteristics of the slow spontaneous oscillations observed in transcranial NIRS recordings from patients with falciparum malaria showed that the VLF spectral power was decreased in cerebral malaria and increased on recovery. The mechanisms underlying these changes could not be determined in the present study.

The origin of the slow cerebral oscillations is still largely unknown, with vasomotion the most commonly quoted as a cause for the LF oscillations [4, 10, 11], while neurogenic stimulation in vessels with diameter 50–100 μm has been suggested by researchers as the origin of the VLF oscillations [12].

Although certain pathological states, such as Alzheimer’s disease [5], cerebral microangiopathy [6] and cerebral infarction [7], are known to impair particularly the LF oscillations, malaria was found to have an effect exclusively on the VLF oscillations. We therefore assume that the observed changes in severe malaria are mediated through a different mechanism. Low nitric oxide bioavailability [13], hypocapnia [14] and raised concentrations of isoprostanes [8] likely affect the regulation of vascular tone in falciparum malaria. Cerebrovascular resistance has been found to be raised in adults with cerebral malaria displaying normal reactivity to changes in pCO_2_, implying preserved autoregulation [15]. It would be interesting to see if the PSD in a different vascular bed was similar to those recorded transcranially. Autonomic control of haemodynamics has received little attention in severe malaria, but could be disturbed in cerebral malaria. Orthostatic hypotension is common in patients with falciparum malaria and is accompanied by a failure of compensatory reflex cardio-acceleration, implying autonomic nervous system dysfunction [16].

We analysed approximately 328 s worth of data from each patient, although significantly longer recordings were available. Due to the restlessness of patients, several recordings were scattered with motion artefacts and in order to analyse the same length of data for all patients we could use only 328 s (2,048 sampling points). We also acknowledge other concerns, such as the limited power of the Fourier analysis in signals with time-varying frequency content, the fact that the recordings were at baseline only without including some kind of intervention and the possibility that the haemoglobin absorption spectrum might be altered in the presence of the parasite product hemozoin.

Despite these shortcomings, the present study constitutes a good first evaluation on changes in slow cerebral oscillations in patients with severe and cerebral malaria. We demonstrated that application of NIRS in a resource-poor setting is possible owing to the non-invasiveness, portability and low cost of this technology and is a promising research tool.
